# CRISPR/Cas9-Mediated Constitutive Loss of VCP (Valosin-Containing Protein) Impairs Proteostasis and Leads to Defective Striated Muscle Structure and Function In Vivo

**DOI:** 10.3390/ijms23126722

**Published:** 2022-06-16

**Authors:** Philipp Voisard, Federica Diofano, Amelia A. Glazier, Wolfgang Rottbauer, Steffen Just

**Affiliations:** 1 Molecular Cardiology, Department of Internal Medicine II, University of Ulm, Albert-Einstein-Allee 23, 89081 Ulm, Germany; philipp.voisard@uni-ulm.de (P.V.); federica.diofano@uniklinik-ulm.de (F.D.); amelia.glazier@uniklinik-ulm.de (A.A.G.); 2 Department of Internal Medicine II, University of Ulm, Albert-Einstein-Allee 23, 89081 Ulm, Germany; wolfang.rottbauer@uniklinik-ulm.de

**Keywords:** VCP, zebrafish, CRISPR/Cas9, VCPopathies, protein homeostasis, disease modeling

## Abstract

Valosin-containing protein (VCP) acts as a key regulator of cellular protein homeostasis by coordinating protein turnover and quality control. Mutations in VCP lead to (cardio-)myopathy and neurodegenerative diseases such as inclusion body myopathy with Paget’s disease of the bone and frontotemporal dementia (IBMPFD) or amyotrophic lateral sclerosis (ALS). To date, due to embryonic lethality, no constitutive VCP knockout animal model exists. Here, we generated a constitutive CRISPR/Cas9-induced *vcp* knockout zebrafish model. Similar to the phenotype of *vcp* morphant knockdown zebrafish embryos, we found that *vcp*-null embryos displayed significantly impaired cardiac and skeletal muscle function. By ultrastructural analysis of skeletal muscle cells and cardiomyocytes, we observed severely disrupted myofibrillar organization and accumulation of inclusion bodies as well as mitochondrial degeneration. *vcp* knockout was associated with a significant accumulation of ubiquitinated proteins, suggesting impaired proteasomal function. Additionally, markers of unfolded protein response (UPR)/ER-stress and autophagy-related mTOR signaling were elevated in *vcp*-deficient embryos, demonstrating impaired proteostasis in VCP-null zebrafish. In conclusion, our findings demonstrate the successful generation of a stable constitutive vcp knockout zebrafish line that will enable characterization of the detailed mechanistic underpinnings of *vcp* loss, particularly the impact of disturbed protein homeostasis on organ development and function in vivo.

## 1. Introduction

Valosin-containing protein (VCP), also known as p97, is a type II AAA ATPase and is critically involved in cellular protein homeostasis by coordinating protein quality control, turnover, and degradation [1,2]. VCP-regulated elimination of misfolded and ubiquitinated proteins is mainly accomplished by two protein degradation machineries: macroautophagy/lysosomal degradation and the ubiquitin-proteasome system (UPS) [3]. Consequently, altered VCP function leads to major disruptions in protein homeostasis. Mutations in VCP are known to cause neuromuscular diseases such as inclusion body myopathy with Paget’s disease of bone and frontotemporal dementia (IBMPFD) [4] and amyotrophic lateral sclerosis (ALS) [5], and also striated muscle diseases such as myofibrillar myopathies (MFMs) [6,7]. Several conditional VCP knockout or loss-of-function models have been developed to dissect tissue-specific VCP-deficiency-mediated molecular pathomechanisms. These models have made valuable contributions to the understanding of VCP pathologies in distinct cell populations and organ systems (i.e., nervous system and skeletal muscle) [8,9,10]. For example, cardiac-restricted VCP loss-of-function has been found to disrupt myofibrillar organization, cause aggregation of myocardial ribosomal proteins, and induce cardiomyopathy [11,12,13]. However, the identified human VCP mutations mainly produce multisystem disorders [14], which necessitates the modeling and investigation of a constitutive, systemic loss of VCP in model organisms. In mice, constitutive systemic VCP knockout resulted in lethality in the peri-implantation stage [15]. Additionally, homozygous VCP loss in the fruit fly is also embryonic lethal, indicating a critical role of VCP during embryogenesis in invertebrate and vertebrate species [16]. Remarkably, we recently found that zebrafish embryos with a systemic but transient Morpholino-modified antisense oligonucleotide-mediated *vcp* knockdown survived up to embryonic day 5 and displayed compromised protein degradation via both the proteasome and the autophagy/lysosome system, which led to severe striated muscle defects during development in vivo [17].

Organogenesis including cardiac and skeletal muscle development in zebrafish is easily assessed due to their embryonic transparency and the fact that embryos can survive up to 5 days post-fertilization without a functional cardiovascular system, since oxygen is available by passive diffusion from the surrounding medium. Here, we generated and characterized a constitutive CRISPR/Cas9-induced zebrafish model of *vcp* deficiency. We found that, similar to *vcp* morphants, CRISPR/Cas9-induced *vcp* knockout embryos are viable until day 3 post-fertilization and demonstrate pronounced heart and skeletal muscle defects due to impaired striated muscle protein homeostasis. Our results introduce a novel systemic *vcp* knockout model that will contribute to the understanding of the pathology and the molecular mechanisms behind, as well as the development of tailored therapeutic options to treat, human *vcp*-induced neuromuscular and striated muscle diseases.

## 2. Results

### 2.1. Targeted Constitutive Knockout of Zebrafish Vcp Leads to Defective Heart and Skeletal Muscle Structure and Function

**Figure 1:**
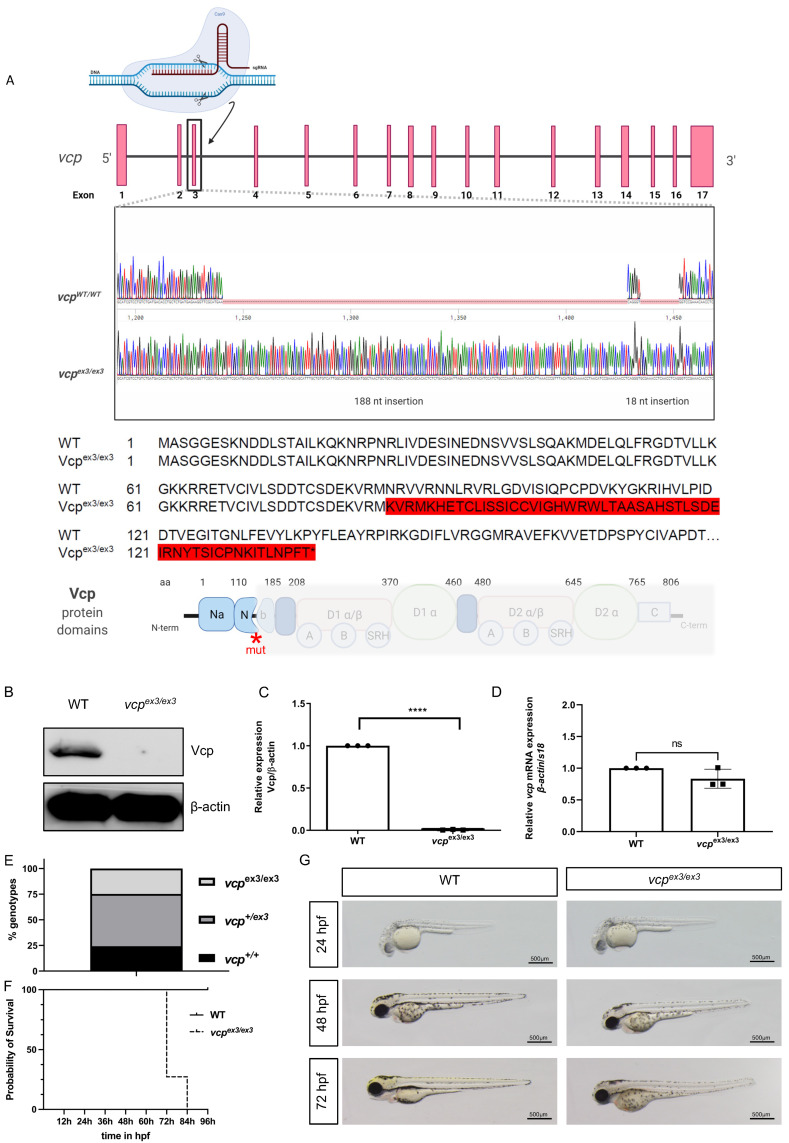
Generation of zebrafish *vcp* knockout by CRISPR/Cas9 gene editing. (**A**) Structure and partial sequence of the *vcp* gene and protein. CRISPR/Cas9 gene editing modifies exon 3 of *vcp* leading to the insertion of 206 nucleotides, a frame shift, and finally the introduction of a premature stop codon, and thereby the premature termination of Vcp translation after 138 amino acids. (**B**,**C**) Immunoblot analysis and quantification of homozygous mutant *vcp*^ex3/ex3^ embryo protein lysate compared to protein lysate obtained from clutchmates (indicated as WT) at 72 hpf (N = 3; mean ± S.D, *p* ≤ 0.0001 determined using two-tailed *t*-test). Error bars indicate s.d.; **** *p* < 0.0001. (**D**) Quantitative real-time PCR of *vcp*^ex3/ex3^ and WT embryos at 72 hpf shows no alterations in *vcp* transcript levels in *vcp*^ex3/ex3^ embryos (N = 3, mean ± SD, *p* < 0.0001 determined using two-tailed *t*-test). Error bars indicate s.d.; ns, not significant. (**E**) In-crosses of heterozygous carriers yielded offspring demonstrating the regular Mendelian genotypic ratio of 25% homozygous wild-type (*vcp^+/+^*; WT), 50% heterozygous (*vcp^+/ex3^*), and 25% homozygous mutant (*vcp*^ex3/ex3^) embryos (N = 3, n = 100). (**F**) A Kaplan–Meier survival curve was constructed for 11 homozygous *vcp*^ex3/ex3^ mutants and 28 control embryos, which showed a sharp increase in mortality starting at 72 hpf in *vcp*^ex3/ex3^ mutants compared to their wild-type clutchmates (n = 39, Mantel–Cox test, *p* < 0.0001). (**G**) Lateral view of brightfield images of WT and *vcp*^ex3/ex3^ zebrafish embryos at 24, 48, and 72 hpf. Homozygous *vcp*^ex3/ex3^ mutants exhibit phenotypic alterations at 72 hpf, whereas *vcp*^ex3/ex3^ mutant embryos at 24 and 48 hpf are indistinguishable from their wild-type clutchmates. ns: not significant.

Constitutive VCP knockout in mice causes early embryonic lethality [15], preventing the investigation of global VCP loss on embryogenesis, organ development, and organ functionality, as well as the assessment of the molecular underpinnings of VCP loss. We recently demonstrated that transient Morpholino-modified antisense oligonucleotide mediated knockdown of *vcp* in zebrafish enabled the mechanistic analysis of *vcp* downregulation during early striated muscle development and function and particularly its impact on protein homeostasis in vivo [17]. Here, we generated and established a systemic CRISPR/Cas9-mediated *vcp* knockout zebrafish line and assessed for the first time the impact of constitutive loss of VCP on striated muscle structure and function in vivo. To do so, we injected crRNA:tracrRNA/Cas9 targeting exon 3 of the zebrafish *vcp* gene into fertilized zebrafish oocytes, recovered an allele defined by a 206-nucleotide insertion (*vcp*^ex3/ex3^) among F1 fish (Figure 1A) and propagated these fish as stable lines at the F3 generation. The mutant allele was verified by PCR followed by gel electrophoresis (Supplementary Figure S1A) and Sanger sequencing. The identified sequence insertion caused the aberrant integration of 52 novel amino acids beginning at amino acid position 85 resulting in a premature stop codon at position 138, with the predicted subsequent loss of all functional Vcp protein domains (Figure 1A). To assess whether the insertional *vcp*^ex3/ex3^ mutation resulted in reduced Vcp protein levels, we performed Western blot analyses and found that levels of Vcp protein in homozygous *vcp*^ex3/ex3^ mutants were below the threshold for detection (N = 3, *p* < 0.0001) (Figure 1B,C), demonstrating the successful knockout of *vcp*. Additionally, we performed quantitative RT-PCR analysis using homozygous *vcp*^ex3/ex3^ mutant embryos and found that *vcp* mRNA levels were not dysregulated compared to control embryos (N = 3, *p =* 0.1274) (Figure 1D), suggesting that the identified 206-nucleotide insertion does not activate the nonsense-mediated mRNA decay (NMD) pathway and thereby genetic compensation/transcriptional adaptation as observed in several other CRISPR/Cas9-induced zebrafish knockout lines [18,19]. The absence of genetic compensation in *vcp*^ex3/ex3^ mutant embryos was further supported by the identification of phenotypic characteristics in *vcp*^ex3/ex3^ mutants that resemble the striated muscle defects observed after the injection of *vcp*-specific Morpholino-modified antisense oligonucleotides or the pharmacological inhibition of Vcp function in zebrafish [17]. Phenotyping and subsequent genotyping of offspring derived from intercrossing adult heterozygous *vcp*^ex3/+^ fish revealed the expected Mendelian ratio of 25% homozygous mutant *vcp*^ex3/ex3^, 50% heterozygous *vcp*^ex3/+^, and 25% homozygous *vcp*^wt^ embryos, with only homozygous mutant *vcp*^ex3/ex3^ embryos showing striated muscle abnormalities at 72 h post fertilization (hpf) (N = 3, n = 100) (Figure 1E). To assess whether wild-type *vcp* mRNA can restore heart and skeletal muscle structure and function in homozygous *vcp*^ex3/ex3^ mutant embryos, we injected *vcp* mRNA into embryos derived from intercrossing heterozygous *vcp*^ex3/ex3^ zebrafish. After injecting 100 pg of wild-type *vcp* mRNA, 76.4% of *vcp*^ex3/ex3^ mutant embryos showed complete rescue of striated muscle structure and function (Supplementary Figure S1B), demonstrating that the CRISPR/Cas9-mediated Vcp deficiency is specific and causative for the heart and skeletal muscle pathology in *vcp*^ex3/ex3^ mutant embryos. A Kaplan–Meier survival curve was constructed for homozygous *vcp*^ex3/ex3^ mutants (n = 11) and control embryos (n = 29), which showed a sharp increase in mortality at 72 hpf in *vcp*^ex3/ex3^ mutants compared to their phenotypically wild-type clutchmates. At 84 hpf, all homozygous *vcp*^ex3/ex3^ mutant embryos were dead, whereas no increased mortality was observed in control embryos (Figure 1F), indicating that the constitutive loss of Vcp results in severe developmental pathologies and lethality. Up to 72 hpf, *vcp*^ex3/ex3^ mutant embryos are indistinguishable from their wild-type clutchmates regarding heart and skeletal muscle structure and function (Figure 1G).

**Figure 2:**
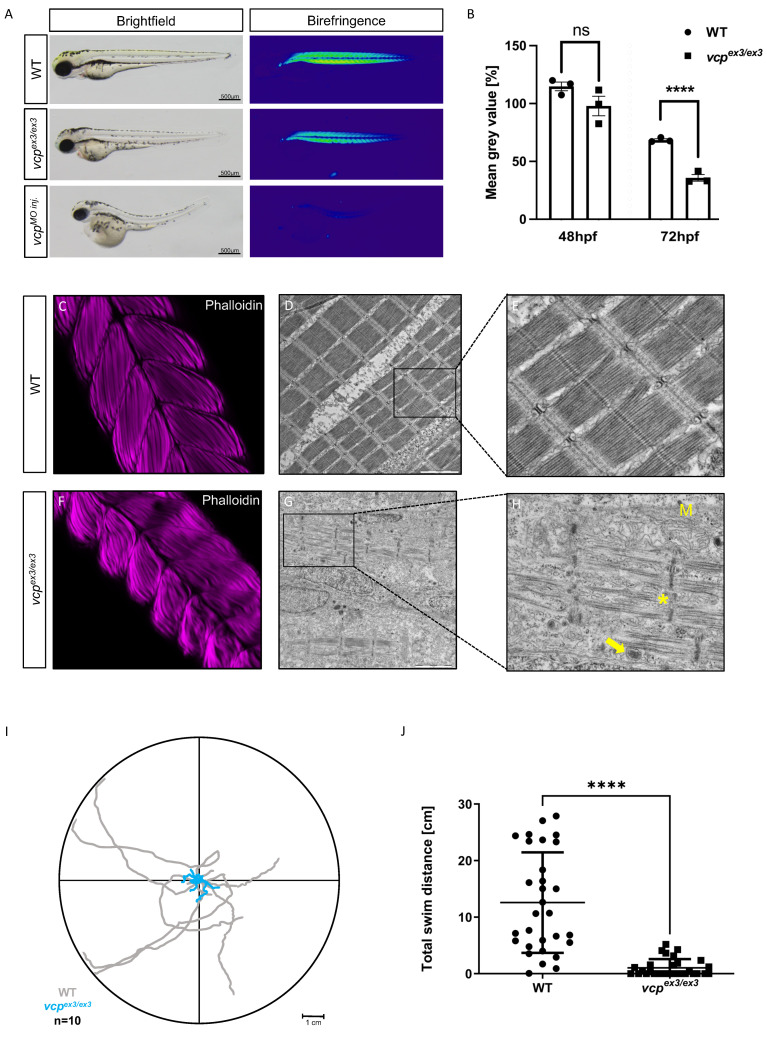
Genetic loss of *vcp* leads to skeletal muscle dysfunctions. (**A**) Lateral view of brightfield and birefringence images for WT, *vcp*^ex3/ex3^, and VCP-MO-injected embryos at 72 hpf. (**B**) Densitometric analysis of the birefringence signal at 72 hpf reveals significantly reduced signal intensity in *vcp*^ex3/ex3^ embryos, implying impaired skeletal muscle organization, whereas birefringence signal intensity is unaltered at 48 hpf (48 hpf: N = 3, n = 5/8, *p* = 0.6690; 72 hpf: N = 3, n = 8/15; *p* = 0.0001; two-tailed *t*-test). Error bars indicate s.d.; **** *p* < 0.0001; ns, not significant. (**C**,**F**) Confocal microscopic analysis of phalloidin-stained skeletal muscles of WT and *vcp*^ex3/ex3^ embryos at 72 hpf. *vcp*^ex3/ex3^ embryos show disrupted muscle fibers compared to the clutchmates. (**D**,**E**,**G**,**H**) Electron microscopic pictures of WT and *vcp*^ex3/ex3^ skeletal muscle embryos at 72 hpf. *vcp*^ex3/ex3^ embryos show damaged mitochondria (M) and increased electron-dense inclusion bodies (→) in addition to the disrupted muscle fibers (*). (**I**) Touch-evoked assay reveals reduction in responsiveness upon mechanical stimulus in *vcp*^ex3/ex3^ embryos, as shown in the motion trace diagram. (**J**) Quantification of the total swim distance shows significantly reduced motility in *vcp*^ex3/ex3^ compared to WT embryos (N = 3, n = 10, mean ± S.D; *p* < 0.0001; two-tailed *t*-test). Error bars indicate s.d.; **** *p* < 0.0001. ns: not significant.

Next, to characterize the observed striated muscle defects in *vcp*^ex3/ex3^ mutants in more detail, we first performed birefringence analysis to assess skeletal muscle organization. Due to the ability of highly organized striated muscle tissue to polarize light, myofibrillar disorganization within the skeletal muscle can be easily visualized by a reduction in birefringence signal intensity [20]. At 48 hpf, in *vcp*^ex3/ex3^ mutant embryos, the birefringence signal from the skeletal muscle was unaltered compared to control embryos (N = 3, *p =* 0.6690), whereas at 72 hpf *vcp*^ex3/ex3^ embryos showed a significant reduction in birefringence signal intensity similar to Vcp morphant embryos and in contrast to control embryos (N = 3, *p =* 0.0001) (Figure 2A,B), implying a disruption of the myofibrillar array [17]. To further support the *vcp*-deficiency-dependent disorganization of skeletal muscles in *vcp*^ex3/ex3^ mutant embryos, we conducted immunohistological analyses as well as transmission electron microscopic (TEM) studies. Whole-mount staining of *vcp*^ex3/ex3^ mutant skeletal muscle tissue with phalloidin revealed myofibers to be less organized compared to clutchmates (Figure 2C,F). As demonstrated by TEM analyses, skeletal muscle cells of wild-type control embryos were densely packed with sarcomeres, composed of highly organized arrays of thick and thin filaments, which are flanked by Z-disks (Figure 2D,E). By contrast, *vcp*^ex3/ex3^ skeletal muscle showed significantly fewer and disorganized myofibrils and sarcomeres (Figure 2G,H). Furthermore, *vcp*^ex3/ex3^ mutant embryos exhibited dysmorphic mitochondria with reduced numbers of cristae as well as an accumulation of inclusion bodies (Figure 2G,H). These findings are consistent with the skeletal muscle pathologies observed after Morpholino-mediated Vcp knockdown [17]. In addition, we assessed the touch-evoked escape response (TEER) of the 30 *vcp*^ex3/ex3^ embryos compared to 30 WT embryos and found that *vcp*^ex3/ex3^ embryos showed significantly impaired motility quantified by total swim distance (N = 3, *p* < 0.0001) (Figure 2I,J).

**Figure 3:**
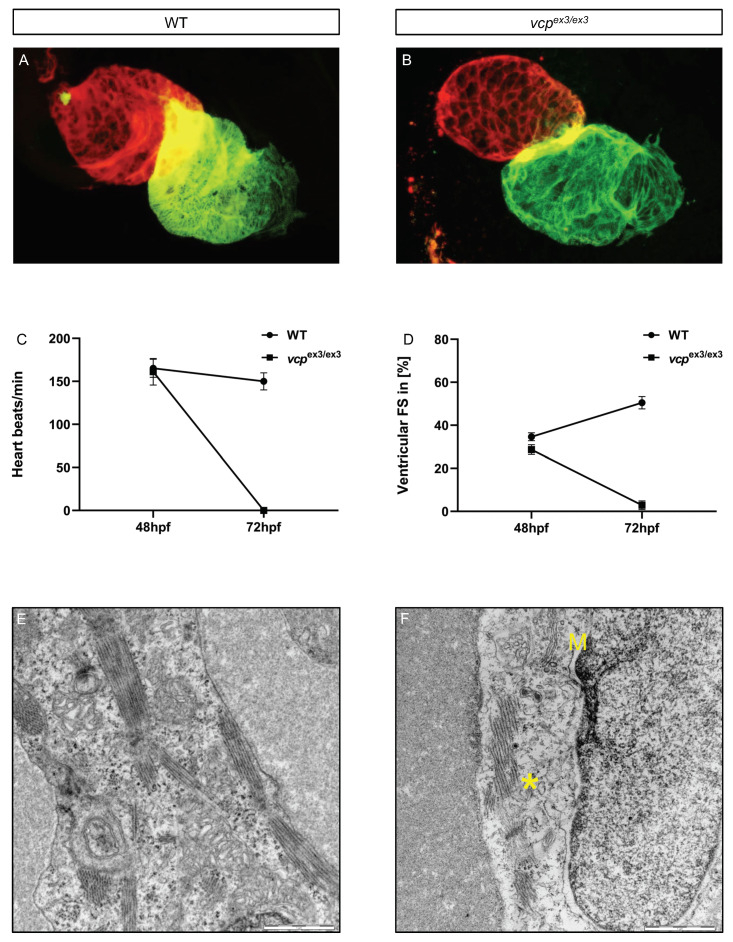
Genetic loss of *vcp* leads to heart failure in homozygous mutant embryos. (**A**,**B**) Analysis of the distribution of meromysin (MF20, green) and atrial-specific myosin heavy chain (S46, red) shows normal expression levels and patterns in *vcp*^ex3/ex3^ embryos, suggesting regular heart chamber specification and cardiomyocyte differentiation. (**C**) Heart rate quantification at 48 hpf does not reveal any alterations in *vcp*^ex3/ex3^ embryos (161 ± 15 heart beat/min) compared to age-matched clutchmates (165 ±10 heart beat/min) (N = 3, n = 11/12, mean ± S.D, *p* = 0.7000 determined using two-tailed *t*-test). At 72 hpf, *vcp*^ex3/ex3^ embryos show highly significant reduction in the heart rate (N = 3, n = 9/12; *vcp*^ex3/ex3^ 0 heart beat/min; WT 150 ± 10 beats/min; mean ± S.D *p* ≤ 0.0001; two-tailed *t*-test). (**D**) Measurement of ventricular fractional shortening at 48 hpf shows no significant differences between WT (34.64 ± 8%) and *vcp*^ex3/ex3^ (28.73 ± 7%) embryos (N = 3, n = 19/9, mean ± S.D; *p* = 0.1000; two-tailed *t*-test). At 72 hpf, fractional shortening is significantly reduced in *vcp*^ex3/ex3^ embryos (N = 3, n = 12, mean ± S.D; FS *vcp*^ex3/ex3^ 3 ± 7%; FS WT 50.48 ± 10%; *p* ≤ 0.0002; two-tailed *t*-test). (**E**,**F**) Electron microscopic pictures of WT and *vcp*^ex3/ex3^ hearts at 72 hpf. *vcp*^ex3/ex3^ embryos show severely dysmorphic mitochondria (M) and disorganized myofibrils (*). ns: not significant.

Next, we analyzed the impact of the CRISPR/Cas9-mediated loss of *vcp* on heart structure and function. We first evaluated the expression of myosin heavy chain isoenzymes that are expressed in a typical heart-chamber-specific pattern [21]. We observed normal distribution of myosin heavy chain (MF20) and the atrial-specific myosin heavy chain (S46) in *vcp*^ex3/ex3^ mutant embryos (Figure 3A,B), indicating that *vcp* loss does not interfere with chamber specification and cardiomyocyte differentiation. Next, we calculated ventricular fractional shortening (FS) in addition to heart rate (HR) to assess cardiac function at 48 and 72 hpf. At 48 hpf, heart rate and fractional shortening in *vcp*^ex3/ex3^ mutants was comparable to wild-type control hearts (HR WT 165 ± 10 beats/min; HR *vcp*^ex3/ex3^: 161 ± 15 beats/min; n = 11/12) (FS WT: 34.64 ± 8%; FS *vcp*^ex3/ex3^: 28.73 ± 7%; n = 19/9) (Figure 3C,D). By contrast, at 72 hpf, we found cardiac function was completely abolished in *vcp*^ex3/ex3^ mutant embryos compared to wild-type clutchmates (HR WT: 150 ± 10 beats/min; HR *vcp*^ex3/ex3^: 0 ± 0 beats/min; n = 9/12) (FS WT: 50.48 ± 10%; FS *vcp*^ex3/ex3^: 3 ± 7%; n = 12) (Figure 3C,D; movies 1–4), suggesting that the constitutive knockout of *vcp* in zebrafish interferes with cardiac function similar to findings in *vcp* morphant embryos [17].

To assess whether defective cardiac function in *vcp*^ex3/ex3^ mutants might be caused by ultrastructural abnormalities, as observed in *vcp*^ex3/ex3^ skeletal muscle cells, we performed TEM studies of *vcp*^ex3/ex3^ mutant cardiomyocytes at 72 hpf. The cardiomyocyte cytoarchitecture of wild-type control embryos appeared normal. Sarcomeres were well-organized, mitochondria were composed of numerous cristae, and no inclusion bodies were present in wild-type cardiomyocytes (Figure 3E). In contrast, *vcp*^ex3/ex3^ mutant cardiomyocytes exhibited reduced numbers of properly organized sarcomeric structures, dysmorphic mitochondria with massively reduced cristae numbers, and inclusion bodies (Figure 3F). These findings suggest that pathologically altered striated muscle ultrastructure accounts for the functional heart and skeletal muscle defects in CRISPR/Cas9-mediated *vcp* knockout zebrafish embryos.

### 2.2. Loss of Vcp Interferes with Protein Homeostasis in Developing Zebrafish

**Figure 4:**
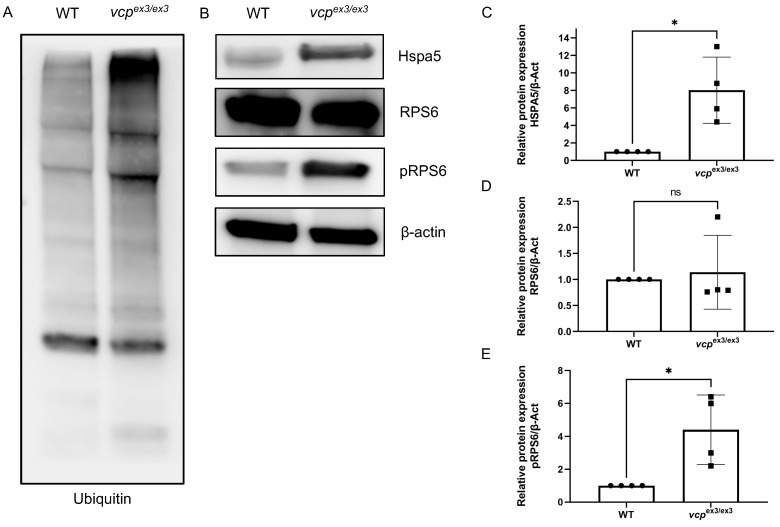
ER stress and impaired protein homeostasis in *vcp*^ex3/ex3^ embryos. (**A**) Loss of Vcp leads to an accumulation of ubiquitinated proteins in *vcp*^ex3/ex3^ embryos compared to WT controls (N = 4). (**B**,**C**) Significantly increased Hspa5 protein levels suggest ER stress in *vcp*^ex3/ex3^ embryos (N = 4; *p* = 0.0286; two-tailed *t*-test). Error bars indicate s.d.; * *p* < 0.05. (**B**,**D**) Total RPS6 protein levels are unchanged in *vcp*^ex3/ex3^ mutant embryos (N = 4; *p* = 0.0286; two-tailed *t*-test), whereas phosphoRPS6 protein levels. Error bars indicate s.d.; ns, not significant. (**B**,**E**) are significantly increased, suggesting an activation of mTORC1 signaling (N = 4; *p* = 0.0286; two-tailed *t*-test). ß-Actin was used as a loading control. Error bars indicate s.d.; * *p* < 0.05. ns: not significant.

We previously found that transient Morpholino-mediated knockdown of *vcp* resulted in compromised protein degradation via both the proteasome (UPS) and the autophagy machinery [17,22]. Hence, to investigate the effects of constitutive and stable *vcp* knockout on protein quality control and degradation, we assessed specific cellular pathways involved in protein homeostasis and known to be regulated by Vcp function. To do so, we investigated the unfolded protein response (UPR)/ER-stress, mTOR signaling, and protein ubiquitination by comparing specific marker protein levels in *vcp*^ex3/ex3^ mutants and control embryos. Similar to molecular patterns in *vcp* morphant zebrafish embryos, we also found significantly elevated poly-ubiquitinated protein levels in *vcp*^ex3/ex3^ zebrafish compared to wild-type clutchmates (Figure 4A), suggesting impaired UPS function caused by the constitutive knockout of *vcp* in vivo. Furthermore, upregulation of the UPR due to ER stress leads to an accumulation of heat-shock proteins (HSPs) such as HSPA5 (the ER-resident Hsp70 chaperone) [23]. We quantified HSPA5 protein levels in *vcp*^ex3/ex3^ mutants and found a significant increase in Hspa5 expression compared to wild-type controls (N = 4; *p =* 0.0286) (Figure 4B,C), suggesting pronounced ER stress in *vcp*^ex3/ex3^ mutant embryos. Finally, mTOR (mechanistic target of rapamycin) dysfunction has been identified as a contributing factor in human diseases such as amyotrophic lateral sclerosis (ALS), which was found to be associated with VCP mutations [24]. mTOR is also a major regulator of autophagy, a process critically controlled by VCP function [24,25,26]. To analyze the impact of *vcp* deficiency on mTOR signaling, we quantified the phosphorylation of ribosomal protein S6 (pRPS6), a downstream mTOR target and molecular mTOR signaling biomarker [27]. Elevated levels of phosphorylated RPS6 are correlated with increased activity of the mTORC1 (mechanistic target of rapamycin complex 1) pathway. Under physiological conditions, Vcp is known to inhibit mTORC1 signaling [28,29,30]. Interestingly, we found significant upregulation of phosphorylated RPS6 (pRPS6) (Figure 4B,E) (N = 4; *p =* 0.0286) in *vcp*^ex3/ex3^ mutant embryos compared to controls, whereas total RPS6 protein levels were unchanged in *vcp*^ex3/ex3^ mutant embryos (Figure 4B,D) (N = 4; *p =* 0.3143), suggesting activated mTOR signaling and thereby the repression of autophagy [31].

In summary, we introduce here a stable, constitutive Vcp knockout model that shows severe heart and skeletal muscle pathologies due to defective protein homeostasis in vivo.

## 3. Discussion

In humans, autosomal-dominant mutations in the *VCP* gene cause severe multisystem degenerative diseases such as inclusion body myopathy associated with Paget’s disease of the bone and frontotemporal dementia (IBMPFD), amyotrophic lateral sclerosis (ALS), Parkinson’s disease (PD), or myofibrillar myopathies (MFMs) [4,5,6,7,32]]. The type II AAA+-ATPase VCP is evolutionary highly conserved and plays a critical role in protein quality control and degradation predominantly by regulating two cellular proteostasis machineries, the ubiquitin-proteasome system (UPS) and the autophagy/lysosome system [1,2,3]. Due to the high complexity of VCP-mediated pathologies and molecular pathomechanisms, animal model systems to investigate and decipher VCP-associated disease mechanisms are fundamental to pave the way for the development of tailored therapeutic treatment options.

Here, we characterized the in vivo impact of constitutive CRISPR/Cas9-mediated Vcp knockout in the vertebrate model system zebrafish and found severe functional and structural striated muscle pathologies in Vcp-null fish. By contrast, global VCP knockout in mice and also in the invertebrate model drosophila was shown to lead to very early embryonic lethality, preventing the investigation of VCP loss on organogenesis during development but also on organ function and protein homeostasis at later developmental stages. Recently, we found that targeted knockdown of Vcp in zebrafish by using Morpholino-modified antisense oligonucleotides led to the progressive loss of cardiac and skeletal muscle structure and function due to significantly impaired protein degradation by the ubiquitin-proteasome and autophagy systems [17]. Similarly, global CRISPR/Cas9-mediated loss of Vcp in zebrafish also resulted in compromised protein homeostasis and striated muscle architecture and function, confirming the Vcp knockdown data and establishing our CRISPR/Cas9-Vcp null zebrafish as a valuable model system to study cell and tissue autonomous and non-autonomous consequences in VCPopathies.

IBMPFD is a degenerative multisystem proteinopathy with severe impact on the brain, bone, and striated muscle including the heart [33]. Due to the embryonic lethality of Vcp loss in mice, transgenic mice overexpressing relevant VCP mutations or conditional, tissue-specific knockout animals were generated and characterized to study VCP-associated pathomechanisms. For instance, the cardiac-specific overexpression of the VCP mutation K524A, which impairs VCP ATPase activity, was recently found to cause severe cardiomyopathy due to a dysregulated endoplasmic-reticulum-associated protein degradation (ERAD) system and significantly compromised protein degradation [11]. Similarly, we also found constitutive CRISPR/Cas9-induced Vcp knockout in zebrafish to cause cardiomyopathy characterized by severely reduced ventricular contractile function. Furthermore, cardiomyopathy was associated with disturbed protein homeostasis as demonstrated by increased markers of UPR/ER stress, an increase in polyubiquitinated proteins and dysregulated autophagy-associated mTOR signaling. Interestingly, cardiac-specific overexpression of wild-type VCP protected mouse hearts against pressure-overload-induced pathological hypertrophy and heart failure [28], substantiating the critical role of VCP in the heart. Moreover, muscle tissue of mice expressing the VCP mutations R155H and A232E, respectively, displayed disrupted myofibrillar structure and mitochondrial degeneration, as well as the accumulation of inclusion bodies [4,34,35]], and haploinsufficient VCP mice manifested with an accumulation of polyubiquitinated proteins caused by impaired UPS function [3,22]. Similar to these findings, we also found compromised skeletal muscle function and structure accompanied impaired myofibrillar organization, dysmorphic mitochondria, and the presence of pathological inclusion bodies.

The relevance of VCP mutations for the onset and the progression of multisystemic degenerative diseases such as IBMPFD, ALS, Parkinson’s disease, or MFMs has been demonstrated by many in vitro and in vivo studies. So far, the constitutive loss of VCP associated with early embryonic lethality has prevented the assessment of the impact of VCP loss and its associated molecular pathomechanisms in an intact physiological context. We established and characterized here a constitutive CRISPR/Cas9-induced Vcp knockout zebrafish line, enabling the investigation of the systemic impact of Vcp loss in vivo. This Vcp knockout model will help to broaden our understanding of Vcp function in health and disease and will hopefully contribute to the development of targeted therapeutic options to treat VCPopathies.

## 4. Materials and Methods

### 4.1. Zebrafish Strains and Injection Procedures

Care and breeding of zebrafish, *Danio rerio,* were conducted as described previously [36]. Fish embryos were staged in hours post fertilization (hpf) as previously described [37]. Movies and pictures were recorded at 48 and 72 hpf. After appropriate institutional approvals (Tierforschungszentrum Ulm University (TFZ), No.1529), the present study was performed conforming to EU Directive 2010/63/EU.

The *vcp^−/−^* zebrafish line was generated using CRISPR-Cas9 technology. A mix consisting of 400 ng/μL recombinant Cas9 protein (Euphoria GmbH, Weimar, Germany), synthetic tracrRNA (100 ng/μL), and gene-specific crRNA (Eurofins Genomics, Ebersberg, Germany) against *vcp* (50 ng/μL) was prepared in 200 mM KCl as described previously [19].

For knocking down *vcp*, we used Morpholino-modified antisense oligonucleotides (MOs) (Gene Tools, LLC) against the splice donor site of exon 2 of *vcp*, as described previously [17]. The Morpholino was injected into 1-cell-stage zebrafish embryos. Sequences of CRISPR RNA oligonucleotides and Morpholinos are summarized in Table S1 in the Supplementary Materials.

Sense-capped *vcp* RNA was synthesized using the mMESSAGE mMASCHINE system (Invitrogen™). An amount of 100 pg RNA was microinjected into 1-cell stage embryos.

### 4.2. Genotyping

Genomic DNA (gDNA) of adult zebrafish tail fins or embryos was extracted using DNA lysis buffer (KOH 1.5 M and EDTA 10 mM). The samples were incubated overnight at 50 °C in 70 μL DNA lysis buffer. The presence of a 206 bp insertion in exon 3 was identified by PCR using the primers described in Table S1. For analyzing PCR products, a 2% agarose gel was used for electrophoresis [38]. Homozygous VCP mutant zebrafish are referred to as *vcp*^ex3/ex3^. Throughout the paper, the description of the clutchmates, consisting of heterozygous *vcp*^ex3/+^ and homozygous *vcp*^wt^ embryos, is referred to as WT.

### 4.3. RNA Extraction and Quantitative Real-Time PCR

A total of 20 *vcp*^ex3/ex3^ embryos and 20 clutchmates (*vcp^+/−^* and *vcp^+/+^*) were collected at 72 hpf for each of the three biological replicates (N = 3) and RNA extraction was carried out using the RNeasy^®^ Mini Kit (Qiagen, Hilden, Germany) according to the manufacturer’s instructions. Reverse transcription was performed using SuperScript^®^ III Reverse Transcriptase (Life Technologies, Waltham, MA, USA), 1 μg total RNA, and oligo(dT) primer. Quantitative real-time PCR was carried out according to standard protocols using SYBR-Green master mix (Roche, Basel, Switzerland) and a Roche LightCycler 480 II. To correct for sample-to-sample variation, housekeeping genes β-actin and 18s ribosomal RNA (18S) were used for normalization [39]. Sequences of primers used are summarized in Table S1.

### 4.4. Immunoblot Analysis

Six *vcp*^ex3/ex3^ zebrafish embryos and six clutchmates were collected at 72 hpf for each of the three or four biological replicates (N = 3–4). Embryos were homogenized and prepared as described by Schnabel et al. [40]. Aliqots of 15 μg protein lysate were solubilized in Laemmli sample buffer and loaded on 8–16% Tris-glycine gels for SDS-PAGE. Electrophoresis was performed using the Mini-PROTEAN Tetra System (BIO-RAD, Hercules, CA, USA). Blotting was performed with the Trans-Blot Turbo System (BIO-RAD), and PVDF membranes were blocked with 5% milk powder in TBST (Tris-buffered saline, 0.05% Tween 20 (Merck, Darmstadt, Germany)) for 1 h at room temperature. After blocking, membranes were incubated with primary antibody (dilution 1:1000) at 4 °C overnight followed by washing and incubating with secondary antibody (dilution 1:2000). Western blots were developed using ECL Prime detection reagent (Cytiva Amersham, Amersham, UK). All membranes were developed using the Pierce ECL Western Blotting Substrate (Thermo Scientific, Waltham, MA, USA) and a luminescent image analyzer (Image Quant Las4000 mini) as described before [39].

Antibodies used: VCP (mouse, #ST1553, Sigma, Burlington, MA, USA), ß-actin (hrp rabbit, #12620, Cellsignal, Danvers, MA, USA), ubiquitin (p4d1, mouse, #3936s, cellsignal), HSPA5 (rabbit, #ab229317, Abcam, Cambridge, UK), phosphorylated RPS6 (rabbit, #22155, cellsignal); sec. abs: HRP-conjugated anti-mouse IgG (#7076, cellsignal), and HRP-conjugated anti-rabbit IgG (#7074, cellsignal).

### 4.5. Light Microscopy Analysis

*vcp*^ex3/ex3^ embryos were collected at 48 and 72 hpf, anaesthetized with tricaine solution, and embedded in 2.5% methylcellulose as previously described [19] for brightfield imaging with an Olympus SZX 16 microscope. Birefringence analysis was performed using two polarizing filters. For data analysis we used Fiji software as previously described [20]. A total of 5 *vcp*^ex3/ex3^ zebrafish embryos and 8 clutchmates were analyzed at 48 and 72 hpf for each of the three biological replicates (N = 3).

### 4.6. Transmission Electron Microscopy

Transmission electron microscopy was performed using zebrafish embryos at 72 hpf. Anaesthetization, embedding, cutting, and contrasting was performed as described previously [41]. Pictures were taken with a JEM-1400 (JOEL USA, Inc., Peabody, MA, USA) electron microscope.

Statistical analysis and generation of graphs were performed with GraphPad Prism 9.1.2, software, San Diego, CA, USA [42].

### 4.7. Immunostaining

Seventy-two-hpf-old WT and *vcp*^ex3/ex3^ embryos were euthanized with tricaine, fixed in 4% paraformaldehyde (PFA) overnight at 4 °C, and incubated with Proteinase K (to allow permeabilization) for 50 min. Samples were washed twice with PBDT (1% BSA, 1% DMSO, 1% Triton X-100 in PBS) and fixed newly in 4% PFA for 20 min. The embryos were washed 5 times with PBDT, blocked for 1 h at room temperature with PBDT enriched with 10% fetal calf serum, and directly stained with Alexa Fluor 568 Phalloidin (1:50, Thermo Fisher Scientific, #A12380) overnight at 4 °C.

Co-immunostaining using the heart muscle myosin heavy chain antibodies MF20 (atrial and ventricular cardiomyocytes) (1:10, mouse monoclonal IgG2b; Hybridomabank, Iowa City, IA, USA) and S46 (atrial cardiomyocytes) (1:50, mouse monoclonal IgG1, Hybridoma Bank) was performed on Dent’s fixed embryos at 72 hpf. Secondary antibodies goat anti-mouse IgG1 Alexa Fluor 488 and Alexa 555 goat anti-mouse IgG2b (both Invitrogen, Waltham, MA, USA) were applied in dilutions of 1:100. Images were acquired using Leica SP8 microscopes (Leica Mikrosysteme Vertrieb GmbH, Wetzlar, Germany).

### 4.8. Touch-Evoked Escape Response Assay (TEER)

A TEER assay was performed as previously described [43] to analyze the motility of zebrafish embryos. E3 medium was maintained at 28 °C in a 14 cm diameter Petri dish (VWR), into which 72 hpf embryos were placed in the center of the dish individually. The embryos were exposed to daylight for 10 min prior to this to allow them to acclimate. After placement, embryos were given 10 s to adjust to the new environment, then touched with a needle at the tip of the caudal fin. From the point of stimulus, a 10 s video was recorded using a digital camera (1080 pixels and 60 frames per second). Distance was calibrated using a ruler placed in the video field of view. Semi-automated motion tracking analysis of the obtained videos was performed with Tracker Video Analysis and Modeling Tool open-source software (physlets.org, accessed on 7 February 2022). The head of each embryo was used as the center of mass. Tracking analysis was used to generate motion trace diagrams, and total swim distance and maximum velocity and acceleration were extracted from motion tracking data.

### 4.9. Cardiac Functional Assessment and Statistical Analysis

Fractional shortening (FS) of zebrafish embryo hearts was determined to estimate contractility and pumping capacity [44]. FS was measured from videos taken using a Leica DM IL LED microscope. Embryos were anesthetized and embedded in 2.5% methyl cellulose at 48 and 72 hpf and the heart was recorded at 20× magnification. Subsequently, the FS was determined by measuring the diameter of the ventricle at the end of the systole and diastole for 6 cardiac cycles per embryo using ImageJ software. Heart beats per minute were counted at 48 and 72 hpf.

Data were analyzed using GraphPad Prism 9 software. All experiments were conducted in minimum biological triplicates (N = 3). All the results are expressed as mean ± standard deviation (S.D.) and statistical analyses were conducted as described in the figure legends. A *p*-value smaller than 0.05 was taken as statistically significant.

## Data Availability

The data presented in this study are available on request from the corresponding author.

## Supplementary Materials

The following supporting information can be downloaded at: https://www.mdpi.com/article/10.3390/ijms23126722/s1.

## References

[1] Van Den Boom J., Meyer H. (2018). VCP/p97-Mediated Unfolding as a Principle in Protein Homeostasis and Signaling. Mol. Cell.

[2] Zhang S.H., Liu J., Kobayashi R., Tonks N.K. (1999). Identification of the cell cycle regulator VCP (p97/CDC48) as a substrate of the band 4.1-related protein-tyrosine phosphatase PTPH1. J. Biol. Chem..

[3] Chen K., Cheng H.H., Zhou R.J. (2012). Molecular mechanisms and functions of autophagy and the ubiquitin-proteasome pathway. Yi Chuan.

[4] Watts G.D.J., Wymer J., Kovach M.J., Mehta S.G., Mumm S., Darvish D., Pestronk A., Whyte M.P., Kimonis V.E. (2004). Inclusion body myopathy associated with Paget disease of bone and frontotemporal dementia is caused by mutant valosin-containing protein. Nat. Genet..

[5] Segawa M., Hoshi A., Naruse H., Kuroda M., Bujo H., Ugawa Y. (2015). A patient with familial amyotrophic lateral sclerosis associated with a new valosin-containing protein (*VCP*) gene mutation. Rinsho Shinkeigaku.

[6] Ferrer I., Olivé M. (2008). Molecular pathology of myofibrillar myopathies. Expert Rev. Mol. Med..

[7] Hübbers C.U., Clemen C.S., Kesper K., Böddrich A., Hofmann A., Kämäräinen O., Tolksdorf K., Stumpf M., Reichelt J., Roth U. (2007). Pathological consequences of VCP mutations on human striated muscle. Brain.

[8] Wani A., Zhu J., Ulrich J.D., Eteleeb A., Sauerbeck A.D., Reitz S.J., Arhzaouy K., Ikenaga C., Yuede C.M., Pittman S.K. (2021). Neuronal VCP loss of function recapitulates FTLD-TDP pathology. Cell Rep..

[9] Arhzaouy K., Papadopoulos C., Schulze N., Pittman S.K., Meyer H., Weihl C.C. (2019). VCP maintains lysosomal homeostasis and TFEB activity in differentiated skeletal muscle. Autophagy.

[10] Wani A., Weihl C.C. (2021). Loss-of-function mutation in VCP mimics the characteristic pathology as in FTLD-TARDBP. Autophagy.

[11] Brody M.J., Vanhoutte D., Bakshi C.V., Liu R., Correll R.N., Sargent M.A., Molkentin J.D. (2019). Disruption of valosin-containing protein activity causes cardiomyopathy and reveals pleiotropic functions in cardiac homeostasis. J. Biol. Chem..

[12] Sun X., Zhou N., Ma B., Wu W., Stoll S., Lai L., Qin G., Qiu H. (2021). Functional Inhibition of Valosin-Containing Protein Induces Cardiac Dilation and Dysfunction in a New Dominant-Negative Transgenic Mouse Model. Cells.

[13] Viswanathan M.C., Blice-Baum A.C., Sang T.K., Cammarato A. (2016). Cardiac-Restricted Expression of VCP/TER94 RNAi or Disease Alleles Perturbs Drosophila Heart Structure and Impairs Function. J. Cardiovasc. Dev. Dis..

[14] Al-Obeidi E., Al-Tahan S., Surampalli A., Goyal N., Wang A.K., Hermann A., Omizo M., Smith C., Mozaffar T., Kimonis V. (2018). Genotype-phenotype study in patients with valosin-containing protein mutations associated with multisystem proteinopathy. Clin. Genet..

[15] Müller J.M., Deinhardt K., Rosewell I., Warren G., Shima D.T. (2007). Targeted deletion of p97 (VCP/CDC48) in mouse results in early embryonic lethality. Biochem. Biophys. Res. Commun..

[16] Higashiyama H., Hirose F., Yamaguchi M., Inoue Y.H., Fujikake N., Matsukage A., Kakizuka A. (2002). Identification of ter94, Drosophila VCP, as a modulator of polyglutamine-induced neurodegeneration. Cell Death Differ..

[17] Kustermann M., Manta L., Paone C., Kustermann J., Lausser L., Wiesner C., Eichinger L., Clemen C.S., Schröder R., Kestler H.A. (2018). Loss of the novel Vcp (valosin containing protein) interactor Washc4 interferes with autophagy-mediated proteostasis in striated muscle and leads to myopathy in vivo. Autophagy.

[18] Sztal T.E., Stainier D.Y.R. (2020). Transcriptional adaptation: A mechanism underlying genetic robustness. Development.

[19] Diofano F., Weinmann K., Schneider I., Thiessen K.D., Rottbauer W., Just S. (2020). Genetic compensation prevents myopathy and heart failure in an in vivo model of Bag3 deficiency. PLoS Genet..

[20] Smith L.L., Beggs A.H., Gupta V.A. (2013). Analysis of skeletal muscle defects in larval zebrafish by birefringence and touch-evoke escape response assays. J. Vis. Exp..

[21] Glickman N.S., Yelon D. (2002). Cardiac development in zebrafish: Coordination of form and function. Semin Cell Dev. Biol..

[22] Imamura S., Yabu T., Yamashita M. (2012). Protective role of cell division cycle 48 (CDC48) protein against neurodegeneration via ubiquitin-proteasome system dysfunction during zebrafish development. J. Biol. Chem..

[23] Hetz C. (2012). The unfolded protein response: Controlling cell fate decisions under ER stress and beyond. Nat. Rev. Mol. Cell Biol..

[24] Hay N., Sonenberg N. (2004). Upstream and downstream of mTOR. Genes Dev..

[25] Ju J.-S., Fuentealba R.A., Miller S.E., Jackson E., Piwnica-Worms D., Baloh R.H., Weihl C.C. (2009). Valosin-containing protein (VCP) is required for autophagy and is disrupted in VCP disease. J. Cell Biol..

[26] Gingras A.C., Raught B., Sonenberg N. (2001). Regulation of translation initiation by FRAP/mTOR. Genes Dev..

[27] Sfakianos A.P., Mellor L.E., Pang Y.F., Kritsiligkou P., Needs H., Abou-Hamdan H., Désaubry L., Poulin G.B., Ashe M.P., Whitmarsh A.J. (2018). The mTOR-S6 kinase pathway promotes stress granule assembly. Cell Death Differ..

[28] Zhou N., Ma B., Stoll S., Hays T.T., Qiu H. (2017). The valosin-containing protein is a novel repressor of cardiomyocyte hypertrophy induced by pressure overload. Aging Cell.

[29] Zhou N., Stoll S., Qiu H. (2017). VCP represses pathological cardiac hypertrophy. Aging (Albany NY).

[30] Shu H., Peng Y., Hang W., Zhou N., Wang D.W. (2021). Emerging role of VCP/p97 in cardiovascular diseases: Novel insights and therapeutic opportunities. Biochem. Soc. Trans..

[31] Rabanal-Ruiz Y., Otten E.G., Korolchuk V.I. (2017). mTORC1 as the main gateway to autophagy. Essays Biochem..

[32] Korb M., Peck A., Alfano L.N., Berger K.I., James M.K., Ghoshal N., Healzer E., Henchcliffe C., Khan S., Mammen P.P.A. (2022). Development of a standard of care for patients with valosin-containing protein associated multisystem proteinopathy. Orphanet J. Rare Dis..

[33] Kimonis V.E., Fulchiero E., Vesa J., Watts G. (2008). VCP disease associated with myopathy, Paget disease of bone and frontotemporal dementia: Review of a unique disorder. Biochim. Biophys. Acta.

[34] Tresse E., Salomons F.A., Vesa J., Bott L.C., Kimonis V., Yao T.P., Dantuma N.P., Taylor J.P. (2010). VCP/p97 is essential for maturation of ubiquitin-containing autophagosomes and this function is impaired by mutations that cause IBMPFD. Autophagy.

[35] Nalbandian A., Llewellyn K.J., Kitazawa M., Yin H.Z., Badadani M., Khanlou N., Edwards R., Nguyen C., Mukherjee J., Mozaffar T. (2012). The homozygote VCP(R¹⁵⁵H/R¹⁵⁵H) mouse model exhibits accelerated human VCP-associated disease pathology. PLoS ONE.

[36] Bühler A., Kustermann M., Bummer T., Rottbauer W., Sandri M., Just S. (2016). Atrogin-1 Deficiency Leads to Myopathy and Heart Failure in Zebrafish. Int. J. Mol. Sci..

[37] Kimmel C.B., Ballard W.W., Kimmel S.R., Ullmann B., Schilling T.F. (1995). Stages of embryonic development of the zebrafish. Dev. Dyn..

[38] Alexandre-Moreno S., Bonet-Fernández J.-M., Atienzar-Aroca R., Aroca-Aguilar J.-D., Escribano J. (2021). Null cyp1b1 Activity in Zebrafish Leads to Variable Craniofacial Defects Associated with Altered Expression of Extracellular Matrix and Lipid Metabolism Genes. Int. J. Mol. Sci..

[39] Hirth S., Bühler A., Bührdel J.B., Rudeck S., Dahme T., Rottbauer W., Just S. (2016). Paxillin and Focal Adhesion Kinase (FAK) Regulate Cardiac Contractility in the Zebrafish Heart. PLoS ONE.

[40] Schnabel D., Castillo-Robles J., Lomeli H. (2019). Protein Purification and Western Blot Detection from Single Zebrafish Embryo. Zebrafish.

[41] Kessler M., Berger I.M., Just S., Rottbauer W. (2015). Loss of dihydrolipoyl succinyltransferase (DLST) leads to reduced resting heart rate in the zebrafish. Basic Res. Cardiol..

[42] Buhrdel J.B., Hirth S., Kessler M., Westphal S., Forster M., Manta L., Wiche G., Schoser B., Schessl J., Schroder R. (2015). In vivo characterization of human myofibrillar myopathy genes in zebrafish. Biochem. Biophys. Res. Commun..

[43] Bose P., Armstrong G.A.B., Drapeau P. (2019). Neuromuscular junction abnormalities in a zebrafish loss-of-function model of TDP-43. J. Neurophysiol..

[44] Benslimane F.M., Zakaria Z.Z., Shurbaji S., Abdelrasool M.K.A., Al-Badr M.A.H.I., Al Absi E.S.K., Yalcin H.C. (2020). Cardiac function and blood flow hemodynamics assessment of zebrafish (Danio rerio) using high-speed video microscopy. Micron.

