# Supplementary material for: CRISPR/Cas9-Mediated Constitutive Loss of VCP (Valosin-Containing Protein) Impairs Proteostasis and Leads to Defective Striated Muscle Structure and Function In Vivo
Source: Int J Mol Sci. 2022 Jun 16;23(12):6722. doi: 10.3390/ijms23126722 (PMC9223409; doi:10.3390/ijms23126722)
Supplement: Supplementary file 1 [file ijms-23-06722-s001.zip › S1_Table.pdf]

Table S1

	Sequence (5′-3′)
vcp Exon3 crRNA	GAAGGTTCGCATGAACAGGGTGG
uni-tracrRNA [1]	AAACAGCAUAGCAAGUUAAAAUAAGGCUAG UCCGUUAUCAACUUGAAAAAGUGGCACCGAGUCGGUGCU
*vcp*_ex3_fwd	AGTGCTGCTGAAGGGTAAGAAG
*vcp*_ex3_rev	ACCTTGAACCAATACCCAGAAA
MO-*vcp* e2i2 splice [2]	TTGACATCCAAAACTGTACCTGAGA
*vcp*_qRT_fwd [3]	GGCGGCTACTAACAGACCTAACA
*vcp*_qRT_rev [3]	GTCAAAGCGCCCAAATCG
*β-actin*_qRT_fwd [4]	CGAGCAGGAGATGGGAACC
*β-actin*_qRT_rev [4]	CAACGGAAACGCTCATTGC
*s18*_qRT_fwd	CACTTGTCCCTCTAAGAAGTTGCA
*s18*_qRT_rev	GGTTGATTCCGATAACGAACGA

References:

1. Kotani, H., K. Taimatsu, R. Ohga, S. Ota and A. Kawahara. "Efficient multiple genome modifications induced by the crrnas, tracrrna and cas9 protein complex in zebrafish." *PLoS One* 10 (2015): e0128319. 10.1371/journal.pone.0128319. https://www.ncbi.nlm.nih.gov/pubmed/26010089.
2. Kustermann, M., L. Manta, C. Paone, J. Kustermann, L. Lausser, C. Wiesner, L. Eichinger, C. S. Clemen, R. Schröder, H. A. Kestler, *et al.* "Loss of the novel vcp (valosin containing protein) interactor washc4 interferes with autophagy-mediated proteostasis in striated muscle and leads to myopathy in vivo." *Autophagy* 14 (2018): 1911-27. 10.1080/15548627.2018.1491491. https://www.ncbi.nlm.nih.gov/pmc/articles/PMC6152520.
3. Mandriani, B., S. Castellana, C. Rinaldi, M. Manzoni, S. Venuto, E. Rodriguez-Aznar, J. Galceran, M. A. Nieto, G. Borsani, E. Monti, *et al.* "Identification of p53-target genes in danio rerio." *Sci Rep* 6 (2016): 32474. 10.1038/srep32474. https://www.ncbi.nlm.nih.gov/pubmed/27581768.
4. McCurley, A. T. and G. V. Callard. "Characterization of housekeeping genes in zebrafish: Male-female differences and effects of tissue type, developmental stage and chemical treatment." *BMC Mol Biol* 9 (2008): 102. 10.1186/1471-2199-9-102. https://www.ncbi.nlm.nih.gov/pubmed/19014500.
