# Supplementary figures and images for: CRISPR/Cas9-Mediated Constitutive Loss of VCP (Valosin-Containing Protein) Impairs Proteostasis and Leads to Defective Striated Muscle Structure and Function In Vivo
Source: Int J Mol Sci. 2022 Jun 16;23(12):6722. doi: 10.3390/ijms23126722 (PMC9223409; doi:10.3390/ijms23126722)

Supplementary

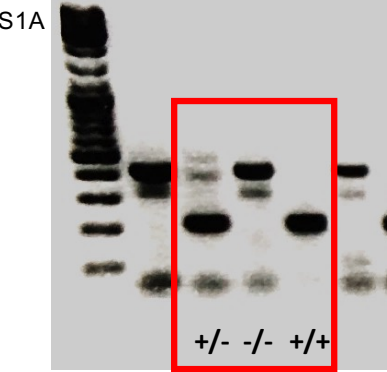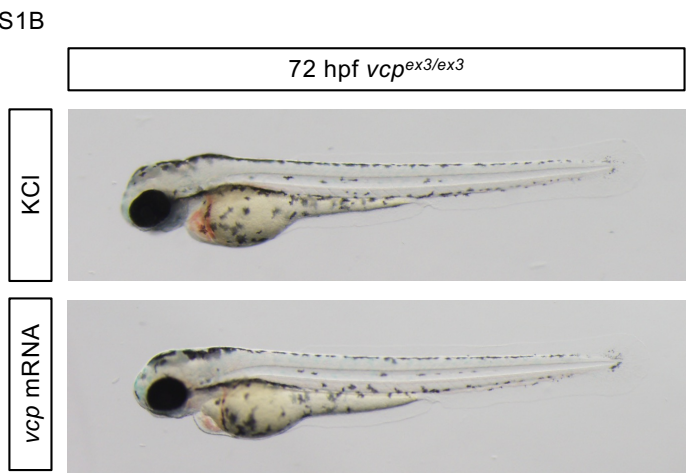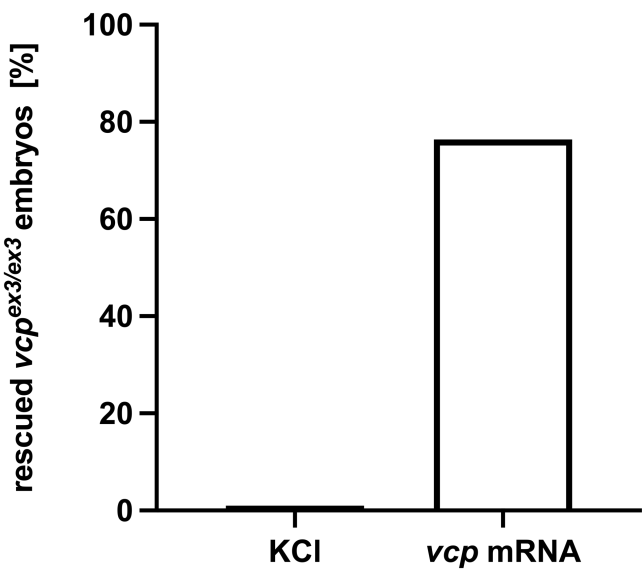

Supplement: Supplementary file 1 [file ijms-23-06722-s001.zip › supplementary.pdf]
